# Defibrillation testing during implantation of the subcutaneous implantable cardioverter-defibrillator: a necessary standard or becoming redundant?

**DOI:** 10.1007/s12471-020-01448-4

**Published:** 2020-08-11

**Authors:** W. van der Stuijt, A. B. E. Quast, R. E. Knops

**Affiliations:** Department of Cardiology, Heart Center, Amsterdam Cardiovascular Sciences, Amsterdam UMC, University of Amsterdam, Amsterdam, The Netherlands

**Keywords:** Implantable defibrillator, Defibrillation testing, Safety margin testing, Ventricular fibrillation, Sudden cardiac death

## Abstract

Since the publication of the SIMPLE and NORDIC trials, defibrillation testing (DFT) is rarely performed during routine implantation of transvenous implantable cardioverter-defibrillators (ICD). However, the results of these trials cannot be extrapolated to the later introduced subcutaneous ICD (S-ICD) and a class I recommendation to perform DFT during the implantation of these devices remains in the current guidelines. Due to the high conversion success rate of DFT on one hand, and the risk of complications on the other, a significant number of physicians omit DFT in S‑ICD recipients. Several retrospective analyses have assessed the safety of the omission of DFT and report contradicting results and recommendations. It is known that implant position, as well as device factors and patient characteristics, influence defibrillation success. A better comprehension of these factors and their relationship could lead to more reliable and safer alternatives to DFT. An ongoing randomised clinical trial, which is expected to end in 2023, is the first study to implement a method that assesses implant position to identify patients who are likely to fail their DFT.

## Dutch contribution to the field

- Amsterdam UMC, location AMC is the sponsor of the PRAETORIAN-DFT trial, which is an international multicentre trial.
- The PRAETORIAN score and the computer modelling study it was designed after were both a production of a Dutch S‑ICD research group.
- The finding that troponin release after defibrillation testing in transvenous ICDs is associated with active lead fixation instead of the defibrillation shocks was first reported by a Dutch S‑ICD research group.
- Step-down defibrillation testing in the S‑ICD was a publication by a Dutch S‑ICD research group.

## Introduction

The implantable cardioverter-defibrillator (ICD) is standard of care for patients with potentially life-threatening ventricular arrhythmias and those at high risk of sudden cardiac death [1, 2]. Defibrillation testing (DFT) has traditionally been part of the implant procedure of the ICD to confirm adequate defibrillation and appropriate sensitivity of the device. This used to be performed through step-down protocols determining the actual defibrillation threshold, and in the beginning these tests were even periodically repeated to ensure an appropriate safety margin. This has evolved into the common clinical practice where a single shock conversion test is performed to ensure adequate device functionality. In transvenous ICDs, adhering to a safety margin of 10J is highly recommended, although physicians may deviate from this practical guideline in certain cases. The most accepted definition defines success of DFT as an ICD shock that successfully terminates an induced ventricular arrhythmia. The landmark trials SIMPLE and NORDIC[3, 4] demonstrated omission of routine DFT to be non-inferior to standard of care with regard to arrhythmic death and first shock efficacy in transvenous ICDs. Although the non-inferiority margin in both studies was quite different, both conclude that a no testing strategy should be preferred because of the risk of complications and lack of benefit from DFT. The omission of routine DFT during routine implantation of left-sided transvenous ICDs is since included in current guidelines with a class IIa recommendation. Except for certain patient populations which are considered to have a high risk of an elevated defibrillation threshold, DFT is now rarely performed. Implantations during which DFT is still recommended include patients with hypertrophic cardiomyopathy or arrhythmogenic right ventricular cardiomyopathy, but also right-sided transvenous ICD implantations.

As the subcutaneous ICD (S-ICD) has become a safe and feasible alternative to the transvenous ICD, the issues of routinely performing DFT during implantation have once again become an issue. The results of the abovementioned landmark trials cannot simply be extrapolated to the later introduced S‑ICD. In the SIMPLE trial, patients were only randomised to either DFT strategy when adequate positioning of the device was confirmed, which was done by adequate sensory signals, an R‑wave of at least 5.0 mV, and an acceptable high-voltage impedance. These measurements established appropriate sensing of the transvenous device and functioned as a safety net whilst omitting DFT in patients randomised to this strategy. However, the sensing characteristics of the S‑ICD are completely modified because of its extrathoracic position. While the intracardiac signal of a transvenous system resembles an intracardiac electrogram, the subcutaneous signal is similar to the surface electrocardiogram. This morphology-based classification only uses one vector, as opposed to the bipolar sensing or dual-coil the transvenous ICD uses to verify the ventricular arrhythmia. This makes the S‑ICD more prone to oversensing of noise and T waves, but also results in less suitable measurements to confirm appropriate device positioning during implantation. Moreover, as the S‑ICD is implanted using anatomical landmarks, there is a wide range in possible implantation positions, which affects the ability of the device to defibrillate successfully. Therefore, there is still a class I recommendation to perform DFT during the implantation of the S‑ICD (Tab. 1). In this review, we aim to elaborate on the potential benefits and disadvantages of DFT, the factors contributing to defibrillation success in S‑ICD implants and the future perspectives on this topic.

**Table 1:** Overview of the current guidelines on defibrillation testing

**2015 HRS/EHRA/APHRS/SOLAECE expert consensus statement**	
Class I	Defibrillation efficacy testing is recommended in patients undergoing a subcutaneous ICD implantation (level C)
Class IIa	It is reasonable to omit defibrillation efficacy testing in patients undergoing initial left pectoral transvenous ICD implantation procedures where appropriate sensing, pacing, and impedance values are obtained with fluoroscopically well-positioned RV leads (level B) Defibrillation efficacy testing is reasonable in patients undergoing right pectoral transvenous ICD implantation or ICD pulse generator changes (level B)
Class III	Defibrillation efficacy testing at time of implantation of a transvenous ICD should not be performed on patients with a documented non-chronic cardiac thrombus, atrial fibrillation or atrial flutter without adequate systemic anticoagulation, critical aortic stenosis, unstable CAD, recent stroke or TIA, haemodynamic instability, or other known comorbidities associated with poor outcomes (level C)
**Boston Scientific Manual Emblem MRI**	
Class IIb	Defibrillation testing may be conducted with a recommended 15J safety margin

## Rationale of routine DFT during S-ICD implantation

The performance of DFT during S‑ICD implantation is recommended to confirm system integrity and reliable sensing. First, for unexperienced implanters, performing DFT is a quick and straightforward method to ensure device positioning and functionality. Furthermore, technical issues where the device fails to sense ventricular fibrillation (VF) are difficult to recognise before or during implantation without DFT, especially with the limited options to confirm the sensing in the S‑ICD. The shock vector varies with the implant position, and the implanter has a wide range in possible implant positions. Finally, in patients at risk for a high defibrillation threshold, such as those who take amiodarone, or who are diagnosed with hypertrophic cardiomyopathy or arrhythmogenic right ventricular cardiomyopathy, performing DFT may be the only way to guarantee an appropriate safety margin.

## Disadvantages of DFT in S-ICD recipients

It may be undesirable to expose all patients to DFT, since the induction of VF and subsequent defibrillation is associated with various complications, albeit with a low incidence rate [5]. The most common complication associated with the performance of DFT is prolonged resuscitation, which in some cases even results in death. However, also complications related to the required anaesthesia, as well as cerebral stroke and inability to convert, have been reported. Haemodynamically comprising complications are more prevalent in patients with a left ventricular function <30%, which is the case for most cardiovascular ICD patients [6, 7]. Moreover, several studies found that defibrillation shocks may cause myocardial damage, depicted as transient depression of left ventricular function, prolonged asystole and an increase in serum troponin levels [8–14]. The latter, however, may be associated with the active fixation of the endocardial lead rather than the shock itself [15]. Finally, the extra personnel required to perform DFT poses a logistical and financial burden on our healthcare system.

**Fig. 1:**
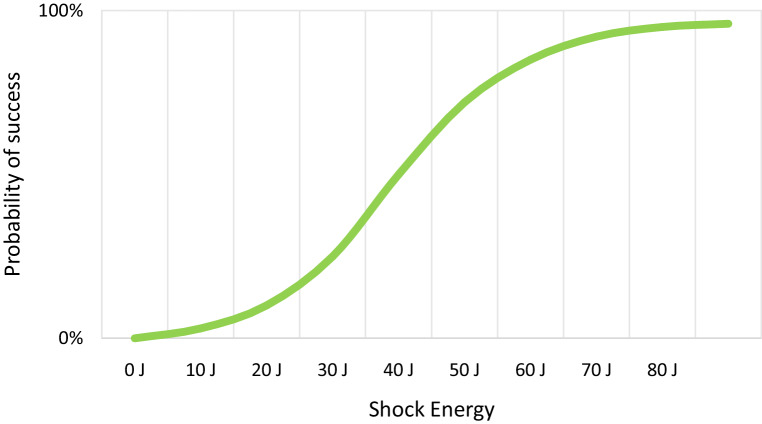
Delivered energy versus probability of successful defibrillation in the S‑ICD

**Fig. 2:**
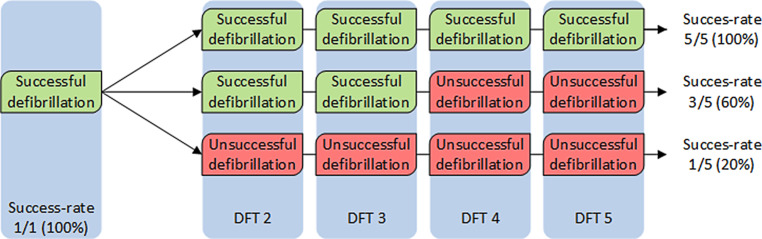
The success-rate of DFT is unpredictable and depends on the amount of tests

Another disadvantage of DFT is the difficult interpretation of the results. There is no threshold in DFT where all shocks above are successful and all shocks below fail. Defibrillation is therefore often described by a probability-of-success curve. The probability of success at a certain shock energy is estimated by the result of a series of defibrillation tests at that same shock energy. This is then repeated at different shock outputs, resulting in a dose-response probability curve, which differs per patient (Fig. 1). Additionally, when a defibrillation shock fails, it is unpredictable whether the next shock at the same shock output will fail too, making the outcome of DFT difficult to appreciate (Fig. 2).

## Comprehension of defibrillation success

Although the defibrillation dose is generally evaluated by the delivered energy of the defibrillator, defibrillation success relies on the amount of electrical current that passes through the myocardium.

**Fig. 3:**
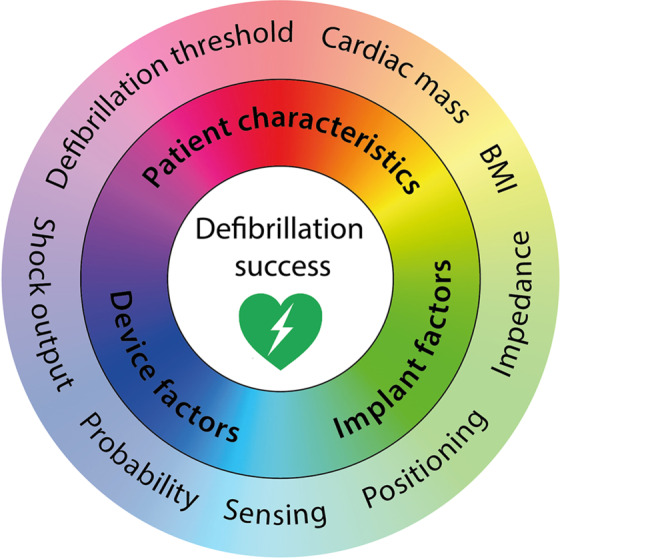
Defibrillation success depends on patients characteristics, device factors and implant factors

This electrical current—and thus defibrillation success—is dependent on several patient characteristics, implant factors and device factors, which also influence each other (Fig. 3). When we acknowledge and understand all these factors, we might be able to safely omit DFT or even predict defibrillation success without testing in the future. Although various factors remain currently unidentified, we describe five factors that are known to influence defibrillation success.

1. *Shock output*
  The electrical current is affected by the shock output of the device. The standard shock output of the S‑ICD is based on the results of DFTs in two pilot studies with temporarily implanted S‑ICDs following a step-down protocol. They aimed to assess the defibrillation threshold, i.e. the minimum shock output that effectively terminated the induced ventricular arrhythmia. The first study enrolled 78 patients and reported an average defibrillation threshold of 32.5 ± 17.0J and the second study reported an average defibrillation threshold of 36.6 ± 19.8J after studying 49 patients [16]. With a safety margin of 15J, a shock output of 80J is deemed safe and effective.
2. *Implant position*
  Recently, a computer model study identified three factors that alter the defibrillation threshold in S‑ICD patients: 1) fat tissue underneath the coil, 2) fat tissue underneath the generator and 3) an anterior placement of the generator on the thoracic wall [17]. When implanting physicians alter their implanting technique with these aspects in mind, step-down testing showed a lower average defibrillation threshold [18]. When implanted intermuscularly with subsequently no fat tissue underneath the generator, another study demonstrated a successful DFT at ≤40J in 94% of the patients [19].
3. *Impedance *
  The current flow is determined not only by the selected shock output, but also by the resistance of the human body. The resistance of the human body between the coil and generator during a shock is represented by the high voltage impedance. As such, patients with high impedance during DFT have a greater chance at DFT failure. However, neither the range nor the determining factors of high voltage impedance of the S‑ICD have been properly evaluated in humans.
4. *Patient characteristics*
  Case reports suggest that the variance in tissue with poor conductive properties, such as fat tissue or emphysematous lung tissue, could explain the range of impedance [20, 21]. Large chest circumference is also a determinant of DFT failure, but this is perhaps solely a consequence of the amount of non-conductive pneumatic tissue [22]. Moreover, cardiac mass and septum thickness are also known to influence defibrillation threshold and defibrillation success [23]. A high body mass index is also associated with a higher risk of defibrillation failure, but this could be related to an inferior implant position with fat tissue underneath the lead or generator.
5. *Device design*
  Device factors also contribute to successful defibrillation. In automatic external defibrillators, it is known that a larger paddle size results in a lower shock impedance, probably resulting in a higher shock efficacy [22]. This could mean that a larger generator or lead surface may also result in a higher shock efficacy, but clinical studies testing this hypothesis are lacking.

## Omission of DFT in S-ICD implants

Regardless of the strong recommendation in the current guidelines, a recent study showed that DFT is only performed in 75% of the patients undergoing de novo S‑ICD implantation in the United States [24]. This low rate is explained by the suggestion that physicians may omit DFT in high-risk patients due to the risk of complications, while simultaneously being resilient with DFT in patients who are likely to have a successful test. No randomised clinical trials have assessed the safety of omitting DFT in S‑ICD recipients yet, but some retrospective analyses have shown diverse outcomes. One analysis showed no difference in mortality between the S‑ICD and transvenous ICD when no DFT was performed during implant [25]. A retrospective comparison between S‑ICD with and without DFT during implant showed no effect on first shock efficacy of the device[26] and a third analysis reported successful defibrillation of all spontaneous ventricular arrhythmias (*n* = 6) after no DFT was performed during implant [27]. On the other hand, one study reported sustained noise oversensing during induced VF in 4% of S‑ICD patients, which resulted in delay of therapy,[28] although this could be the result of oversensing of myopotentials by the diaphragmatic muscles caused by the induction of VF [29]. Another single centre study showed that the first shock during DFT was successful in just 75% and argues that DFT is still necessary for S‑ICD implants [30]. However, these results do not comply with the first published studies about S‑ICD safety, which showed a high conversion success rate of DFT during S‑ICD implants, varying from 98.7–100% [31–34]. Tab. 2 shows an overview of the DFT success rate and first shock efficacy in spontaneous events of the S‑ICD, as reported in recent literature.

**Table 2:** Literature overview of DFT success rate and first shock efficacy in spontaneous events of the S‑ICD

**Author [year of publication] (ref)**	**DFT success rate**	**First shock efficacy in spontaneous events**
Weiss [2013] [34]	100% (304/304)	92.1% (35/38)
Frommeyer [2016] [30]	75% (74/98)	NA
Maurizi [2017] [26]	97.6% (40/41)	NA
Boersma [2017] [31]	99.5% (857/861)	88.5% Unknown
Gold [2017] [33]	98.7% (1394/1412)	NA
Peddareddy [2018] [27]	83.7% (113/135)	88.4% (61/69)
Le Polain de Waroux [2018] [28]	92% (118/128)	NA
Boersma [2019] [32]	99.2% (905/912)	NA

At present, physicians are exploring alternatives to avoid the complications associated with DFT. Some prefer impedance measurements, because the high voltage impedance can be measured with a synchronous 10J shock and without the need for VF induction. However, a low shock impedance may be the result of shunting over the thoracic wall, with no electrical current passing through the myocardium. Therefore, low impedance does not guarantee successful defibrillation, making it unsuited as a surrogate for DFT. Others came up with a method to assess implant position to identify patients who are likely to fail their DFT [35]. Currently, an ongoing randomised controlled trial is testing the hypothesis that S‑ICD implantation is safe when these implant requirements are met: PRAETORIAN-DFT (NCT03495297) [36]. This trial, the results of which are expected in 2023, will be the first study to take the different factors that influence defibrillation success into account.

## Considerations for DFT in special populations

After publication of PRAETORIAN-DFT, it could be that routine DFT in de novo S‑ICD implantations will no longer be recommended by the guidelines. However, in certain populations it remains rational to perform DFT to ensure device functionality. Confirmation of appropriate sensing is especially required in patients whose QRS amplitude has diminished after initial implant and as a consequence are at risk of undersensing of VF, or those who have experienced failed appropriate shocks. Furthermore, in patients with a deviant chest anatomy, such as pectus carinatum, the defibrillation threshold is unpredictable. In morbidly obese patients (body mass index >35 kg/m^2^), there is a higher chance of an inferior implanting position, especially in unexperienced implanters. Although DFT could help to assess the implant position and chance of successful defibrillation, less invasive methods are preferred. Finally, randomised clinical trials in paediatric patients are extremely rare, and it is unlikely that omission of DFT during S‑ICD implant in children will find any scientific basis. However, one could argue that the shock output of 80J combined with the low body mass of children will almost always result in successful defibrillation, possibly making DFT redundant.

## Conclusions

Despite a class I recommendation, many physicians decide to omit DFT in S‑ICD recipients. Indeed, DFT comes with a risk of various complications and the results are difficult to interpret due to probability. With a more thorough comprehension of the patient characteristics, implant factors and device factors that influence defibrillation success, we may be able to safely omit DFT during S‑ICD implants in the future. An ongoing randomised clinical trial, which is expected to end in 2023, is the first study that implements a method that assesses implant position to identify patients who are likely to fail their DFT.
